# The Relationship between Self-Directed Learning and Problem-Solving Ability: The Mediating Role of Academic Self-Efficacy and Self-Regulated Learning among Nursing Students

**DOI:** 10.3390/ijerph18041738

**Published:** 2021-02-11

**Authors:** Younghui Hwang, Jihyun Oh

**Affiliations:** 1 Department of Nursing, University of Ulsan, Ulsan 44610, Korea; hyh77@ulsan.ac.kr; 2 Department of Nursing, Daejeon University, Daejeon 34520, Korea

**Keywords:** self-directed learning, self-regulated learning, self-efficacy, problem-solving, nursing students

## Abstract

Problem-solving ability is necessary for the clinical reasoning and decision-making of nurses to solve patients’ health problems. This study aims to investigate the association between self-directed learning and problem-solving ability using the multiple mediation model to identify strategies to enhance problem-solving ability in nursing students. This is a descriptive survey study of 193 nursing students from two universities in South Korea. Data about self-directed learning, self-regulated learning, academic self-efficacy, and problem-solving ability were collected using structured questionnaires between 5 March and 17 June 2018, and were analyzed using serial multiple mediation analysis. The direct effect of self-directed learning on problem-solving ability was statistically significant. The serial multiple mediation technique predicting problem-solving ability from self-directed learning, academic self-efficacy, and self-regulated learning was significant, explaining 40% of the variance in problem-solving ability. The relationship between self-directed learning and problem-solving ability was partially mediated by academic self-efficacy and self-regulated learning. This study suggests the suitability of considering academic self-efficacy and self-regulated learning together when conducting self-directed learning to improve nursing students’ problem-solving ability.

## 1. Introduction

Problem-solving ability is the skill of identifying a problem and taking action to solve it [1], in particular, it is essential for health professionals to perform clinical reasoning and make a decision [2]. Clinical reasoning is used to identify and explore causal relationships between sets of causes and effects and to achieve a deeper understanding of complex phenomena [3]. Nurses with problem-solving ability can more effectively analyze and research the health problems of patients [3]. Additionally, they can draw effective and targeted solutions that address the root causes of the health problems and devise and execute nursing intervention plans [4].

As the economy and medical technology develop, the number of patients with severe and complex health problems is increasing [5,6]. Human clinical reasoning is not always logical, and people can make mistakes in their reasoning; the more difficult and complex the problems are, the more likely people are to make a wrong decision [3]. However, as nurses with problem-solving ability have personal control capabilities, interest, and willingness, they more easily organize and resolve issues and reduce medical errors [7]. In addition, problem-solving abilities can help nurses develop and implement the most appropriate individualized and holistic care plan [8] and identify any solutions to public health problems using existing resources and a practical approach [4]. Therefore, it is important to improve the problem-solving ability of nursing students in nursing education.

The problem-solving process includes a reflective judgement process that monitors the implementation and results and modifies the strategy/action if necessary [2]. To implement the reflective judgement process, the capacity for self-directed learning is required [2,9]. Self-directed learning can help nursing students in data seeking and analysis of the reflective process [2]. In addition, self-directed learning can help solve problems effectively by providing motivation and learning strategies [10]. Self-directed learning is also the ability to take primary responsibility and control of one’s own learning experience [11]. Therefore, self-directed learning is necessary not only to acquire nursing knowledge in college, but also to perform one’s duties sufficiently and achieve self-development in their career after graduation [12,13].

Many studies have reported that a higher self-directed learning ability is linked to a higher problem-solving ability [12,14]. Therefore, to improve problem-solving ability, education to enhance self-directed learning such as simulation-based education has been provided in nursing education [2]. However, it is not easy to improve problem-solving ability by simply using self-directed learning [15]. Therefore, it is necessary to identify whether another variable such as academic self-efficacy [16] or self-regulated learning [17], known to affect problem-solving ability, can be used to enhance problem-solving ability along with self-directed learning. Self-regulated learning is similar to self-directed learning but has a greater emphasis on constructive and cognitive processes [17]. As self-regulated learning promotes students’ ability to control or manage their learning environment and process with responsibility and belief of the successful performance of tasks [18], self-regulated learning may affect problem-solving ability [17]. Self-efficacy refers to belief in one’s capabilities to successfully execute given tasks [19]. Self-efficacy affects not only decisions regarding learning and knowledge maintenance, but also organizing and implementing learning activities [6]. As self-efficacy is effective to control learning, it can foster self-control. The higher the self-efficacy ability is, the higher the problem-solving ability will be [16].

The successful introduction of self-directed learning into nursing curricula requires an adequate teacher and student readiness [20]. Korean nursing students focus on liberal arts and basic subjects in the first and second years and major subjects and clinical practice in the third year. If self-directed learning is well prepared in the first and second year, it can be conducted well in clinical practice and major subjects in the third-year [20]. However, self-directed learning readiness was found to be low in the first and second year [21]. Therefore, the subjects of this study were first- and second-year nursing students who had to prepare for self-directed learning.

Few studies have used structural causality to determine the effects of self-directed learning, self-regulated learning, and self-efficacy on problem-solving ability. Mediation analysis can identify the effects of a third variable in the pathway between an exposure and an outcome, and multiple mediation analysis enables the consideration of multiple mediators/confounders simultaneously [22]. Accordingly, this study aims to investigate the association between self-directed learning and problem-solving ability based on a multiple mediation model. The relative influence and structural causality of self-directed learning, self-regulated learning, and academic self-efficacy on problem-solving ability in nursing students were examined.

## 2. Methods

### 2.1. Study Design and Participants

This cross-sectional study examined the relationship between self-directed learning and problem-solving ability in first- and second-year nursing students in South Korea. The required sample size was calculated using G power 3.1 [23]. The minimal sample size (*n* = 138) was calculated under the assumptions of a significance level of 0.05, an effect size of 0.15, and a power of 0.95. Therefore, the sample size for this study was adequate. In total, 215 students were approached to participate in this research and 193 completed the questionnaire, giving an overall response rate of 89.7%.

### 2.2. Variables

Problem-solving ability was measured with a tool developed by Lee et al. [24]. The scale comprises 30 items, with five items for each subscale of clarifying the problem, seeking a solution, decision-making, applying the solution, and evaluation and reflection. Each item is rated on a five-point Likert scale from 1 (“very rarely”) to 5 (“very frequently”). A higher score indicates higher problem-solving ability. Cronbach’s α of this tool in Lee et al. [24] was 0.93, and in the present study it was 0.91, indicating high reliability.

Academic self-efficacy was measured using the academic self-efficacy scale developed by Kim and Park [25]. This scale consists of 28 items rated on a five-point Likert scale from 1 (“not at all”) to 5 (“very well”). The scale comprises subscales of self-confidence, task difficulty preference, and self-regulated efficacy in nursing students. A higher score indicates higher academic self-efficacy. Cronbach’s α of this tool in Kim and Park [25] was 0.84, and in the present study it was 0.87, demonstrating acceptable internal consistency.

Self-directed learning was assessed with the Korean self-directed learning ability scale, originally developed by Lee et al. [26]. This scale consists of 45 items in three dimensions of learning planning, leaning practice, and learning evaluation. Each item was scored from 1 (“very rare”) to 5 (“very common”), with a higher score indicating better self-directed learning. Cronbach’s α of this tool in Lee et al. [26] was 0.93, and it was 0.89 in the present study, indicating acceptable internal consistency.

Self-regulated learning strategy was measured using the Motivated Strategies for Learning Questionnaire (MSLQ) originally developed by Pintrich et al. [27] and modified and adapted by Kim [28]. The MSLQ contains 81 items rated on a seven-point scale, ranging from 1 (“not at all true of me”) to 7 (“very true of me”). The questionnaire comprises three domains of cognitive strategies, motivation strategies, and resource management strategies. High scores indicate greater levels of self-regulated learning strategy. Cronbach’s α of this tool in Pintrich et al. [27] was 0.93, equal to that of the present study, indicating high reliability.

### 2.3. Data Collection

The study was conducted between 5 March and 17 June 2018 at two universities in South Korea. Data were collected from first- to second-year nursing students using structured questionnaires. The researchers directly distributed to nursing students who were asked to return the forms to researchers after they had completed them.

### 2.4. Study Analysis

Descriptive statistics and Pearson’s correlation coefficients were analyzed with SPSS statistical software (version 23; IBM Corp, Armonk, New York, USA). To evaluate the direct effects of academic self-efficacy and self-regulated learning as serial mediators of the relationship between self-directed learning and problem-solving ability, SPSS macro PROCESS (model 6) [29] was used for serial multiple mediation analysis. This method explains the direct and indirect effects of self-directed learning (*X)* on problem-solving ability (*Y*) via causally linked multiple mediators of academic self-efficacy (*M*_1_) and self-regulated learning (*M_2_)*. The bootstrap method was used to estimate the 95% bias-corrected confidence interval (BC CI) for each proposed indirect path coefficient and 5000 bootstrap samples [29,30]. For the indirect effect tests, BC CIs that do not include zero indicate significant mediation.

### 2.5. Ethical Considerations

The present study was approved by a University Institutional Review Board (1040968-A-2018-003). All participants were informed of the details of study purpose and procedure and signed a written consent form before the survey was administered. Surveys were directly distributed only to those who consented to study participation. The research emphasized that there was no disadvantage to students if they refused to participate or did not complete the questionnaire. Subjects took approximately 20 min to complete the questionnaire anonymously.

## 3. Results

### 3.1. Descriptive and Bivariate Analyses

Table 1 shows the general characteristics of the participants.

Table 2 presents the levels of all variables. The mean problem-solving ability score was 3.56 (*SD* = 0.45) of 5, higher than the midpoint. The mean self-directed learning and mean academic self-efficacy were 3.46 (*SD* = 0.39) and 3.09 (*SD* = 0.45) of 5, respectively. The mean self-regulated learning score was 4.78 (*SD* = 0.50) of 7, also higher than the midpoint.

Self-directed learning showed significant positive correlations with academic self-efficacy (*r* = 0.554, *p* < 0.001), self-regulated learning (*r* = 0.664, *p* < 0.001), and problem-solving ability (*r* = 0.516, *p* < 0.001). Academic self-efficacy was positively correlated with self-regulated learning (*r* = 0.538, *p* < 0.001) and problem-solving ability (*r* = 0.531, *p* < 0.001). Self-regulated learning was positively correlated with problem-solving ability (*r* = 0.528, *p* < 0.001).

### 3.2. Mediating Effects

**Figure 1:**
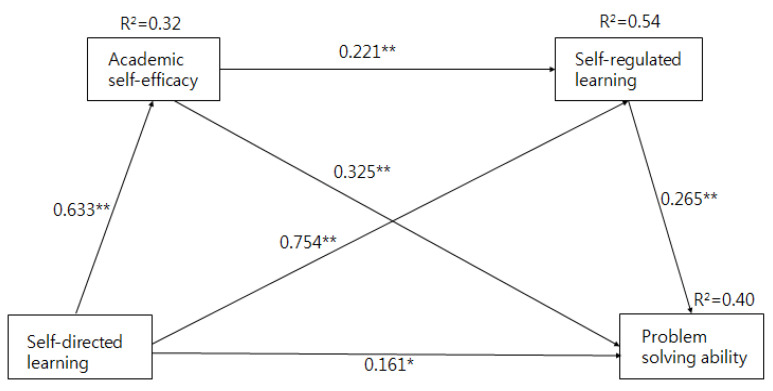
Results of the multiple mediation testing academic self-efficacy and self-regulated learning as mediators of the effect of self-directed learning on problem-solving ability. * p < 0.001. ** *p* < 0.05.

To test the effect of self-directed learning on problem-solving ability through academic self-efficacy and self-regulated learning, serial multiple mediation analysis was performed. The results of the serial multiple mediation analysis are shown in Table 3. Figure 1 illustrates the standardized path coefficients. The effect of self-directed learning on problem-solving ability was increased but significant when the model included self-efficacy and self-regulated learning as mediators. Specifically, the total effect of self-directed learning on problem-solving ability was statistically significant (*B* = 0.585, *SE* = 0.070, *t* = 8.331, *p <* 0.001), as was the direct effect on problem-solving ability (*B* = 0.218, *SE* = 0.091, *t* = 2.397, *p =* 0.017). The effect of serial multiple mediation on predicting problem-solving ability from self-directed learning, academic self-efficacy, and self-regulated learning was significant, explaining 40% of the variance in problem-solving ability (*R^2^* = 0.40, *F* = 39.197, *p* < 0.001).

In serial multiple mediation, the bootstrapping procedure presented two significant indirect effects between self-directed learning and problem-solving ability with a mediating effect of academic self-efficacy (*B* = 0.184, *SE* = 0.048, BC CI [0.093, 0.283]) and self-regulated learning (*B* = 0.037, *SE* = 0.018, BC CI [0.008, 0.077]). Moreover, there was a significant serial mediation between self-directed learning and problem-solving ability via increased academic self-efficacy and sequentially increased self-regulated learning (*B* = 0.145, *SE* = 0.053, BC CI [0.046, 0.258]). Thus, increasing self-directed learning can directly improve problem-solving ability. In sum, these results indicate that the relationship between self-directed learning and problem-solving ability was partially mediated by academic self-efficacy and self-regulated learning.

## 4. Discussion

This study identified the association between self-directed learning and problem-solving ability using the multiple mediation model in nursing students. Based on the results of this study, the relationship between self-directed learning and problem-solving ability seems to be multifactorial.

In this study, problem-solving ability was significantly positively correlated with self-directed learning, and self-directed learning directly affected problem-solving ability. This indicates that the higher the level of self-directed learning of a nursing student is, the higher their problem-solving ability will be; problem-solving ability was promoted by facilitating self-directed learning. This finding supports the previous findings [6,16]. Self-directed learning helps motivate the search for new data to evaluate and modify results achieved in the reflective judgment process [2,31]. For self-directed learning to be successfully implemented, learners should be familiar with the method, and the educational environment must allow proper evaluation of the results of self-directed learning [32]. Unfortunately, such self-directed learning is new in the field and will require time and effort to become established in nursing education [31]. However, to enhance the problem-solving ability of nursing students, self-directed learning is necessary. Therefore, efforts will be needed to develop curricula and teaching methods to establish self-directed learning in nursing education.

It is very important in this study that academic self-efficacy has a mediating effect on the relationship between self-directed learning and problem-solving ability, consistent with the results from Zhang et al. [6]. Academic self-efficacy was significantly positively correlated with self-regulated learning, self-directed learning, and problem-solving ability, consistent with the results from other studies [6,16,31,32]. This indicates that the higher the level of a nursing student’s academic self-efficacy is, the higher the level of self-regulated learning, the level of self-directed learning, and the ability to solve problems will be. In addition, academic self-efficacy was a partial mediator not only in the relationship between self-directed learning and problem-solving ability, but also in the relationship between self-directed learning and problem-solving ability through self-regulated learning. These current study results highlight the importance of academic self-efficacy in improving problem-solving ability using self-directed learning. Problem-based learning and flipped learning, known to improve self-directed learning, have not always been effective in improving the problem-solving ability of nursing students [20,23]. It might be necessary to prepare learners and circumstances for self-directed learning. It may be difficult to apply self-directed learning if learners prefer lecture-oriented education or are familiar with a teacher-centered approach to learning [33]. Therefore, the results suggest that it is important to increase the academic self-efficacy of nursing students when trying to increase problem-solving ability through self-directed learning to make students feel a sense of accomplishment. The experience of success with self-directed learning will help students in self-directed learning, increasing their intention to continue self-directed learning [34]. Nursing students who can exert self-directed learning control over their own learning experience can positively evaluate their ability to conduct and organize academic performance [35]. Nursing students with high academic self-efficacy positively evaluate their ability to solve problems [36]. This could be a driving force to find solutions to difficult and complex problems with confidence, increasing one’s ability to solve problems. Further research needs to develop strategies that can enhance academic self-efficacy to improve problem-solving ability through self-directed learning.

Self-regulated learning was also a partial mediator in the relationship between self-directed learning and problem-solving ability in this study. The result indicates that self-regulated learning can help strengthen nursing students’ ability to control their learning environment and solve problems [17,18]. Self-regulated learning encourages nursing students to believe in their abilities and to motivate and control their learning [18,31]. This indicates that self-regulated learning is a very important ability of nurses to solve complex health problems in the fast-growing and knowledge-incentive healthcare sector [31]. Therefore, in the future, it is necessary to find ways to apply self-regulated learning to establish self-regulated learning in nursing education.

Lastly, when developing a program that improves problem-solving ability, it will be more effective to develop educational methods that can improve academic self-efficacy, self-directed learning ability, and self-regulated learning ability to produce synergic effects.

This study has some limitations. First, the cross-sectional nature of this study limits the ability to draw conclusions about the direction of the relationship among the variables investigated. Therefore, prospective studies are needed to confirm the results. Second, other variables that may affect problem-solving skills have not been considered. Further research will be needed to identify the effects on problem-solving ability using additional variables.

Despite these limitations, this study provides new insight into the relationship between self-directed learning and problem-solving ability, including academic self-efficacy and self-regulated learning. This current study suggests that it is effective to develop academic self-efficacy and self-regulated learning together when conducting self-directed learning to enhance nursing students’ problem-solving ability.

## 5. Conclusions

This study aimed to identify the association between self-directed learning and problem-solving ability using a multiple mediation model. The direct effect of self-directed learning on problem-solving ability was statistically significant. The effect of serial multiple mediation on predicting problem-solving ability from self-directed learning, academic self-efficacy, and self-regulated learning was significant, explaining 40% of the variance in problem-solving ability. The current study results highlight the importance of academic self-efficacy in problem-solving ability and suggest the need for strategies for improving academic self-efficacy. The development of the curriculum and teaching methods for applying self-directed learning to nursing students is needed so that self-directed learning can be continued even after graduation. Continuous efforts will be needed to ensure that self-regulated learning can be established in nursing education. Future research for developing intervention programs might be needed to identify whether using self-efficacy and self-regulated learning is effective when conducting self-directed learning to improve nursing students’ problem-solving ability.

## Figures and Tables

**Table 1:** Descriptive statistics of the study population (*n* = 193).

Variable	*n* (%)
Mean age (years) (SD, range)	19.10 (1.27, 18 to 26)
Gender	
Male	23 (11.9)
Female	170 (88.1)
Grade level	
First year	107 (55.4)
Second year	86 (44.6)
Nursing Major satisfaction	
Dissatisfied	4 (2.1)
Neutral	65 (33.7)
Satisfied	106 (54.9)
Extremely satisfied	18 (9.3)
Academic life satisfaction	
Dissatisfied	6 (3.1)
Neutral	76 (39.4)
Satisfied	94 (48.7)
Extremely satisfied	17 (8.8)

*Note.* SD = Standard Deviation.

**Table 2:** Levels of self-directed learning, academic self-efficacy, self-regulated learning, and problem-solving ability (*n* = 193).

Variables	Min-Max	Mean (SD)
Problem-solving ability	2.40–4.77	3.56 (0.45)
Self-directed learning	2.22–4.56	3.46 (0.39)
Academic self-efficacy	1.71–4.43	3.09 (0.45)
Self-regulated learning	3.46–6.15	4.78 (0.50)

*Note.* SD = Standard Deviation.

**Table 3:** Total, direct, and indirect effects using the multiple mediator model (*n* = 193).

	Effect	SE	*t*	*p*	95% BC CI
Total effect of self-directed learning on problem-solving ability	0.585	0.070	8.331	<0.001	[0.447, 0.724]
Direct effect of self-directed learning on problem-solving ability	0.218	0.091	2.397	0.017	[0.038, 0.485]
Total indirect effect of self-directed learning on problem-solving ability	0.367	0.065			[0.241, 0.499]
Self-directed learning, academic self-efficacy, problem-solving ability	0.184	0.048			[0.093, 0.283]
Self-directed learning, self-regulated learning, problem-solving ability	0.037	0.018			[0.008, 0.077]
Self-directed learning, academic self-efficacy, self-regulated learning, problem-solving ability	0.145	0.053			[0.046, 0.258]

*Note.* BC CI, bias-corrected confidence interval; SE, standard error.

## Data Availability

The data presented in this study are available on request from the corresponding author. The data are not publicly available due to privacy.
